# Morphofunctional study on the effects of passive smoking in kidneys of rats

**DOI:** 10.31744/einstein_journal/2021AO6000

**Published:** 2021-10-25

**Authors:** Carlos Alberto de Moraes, Mercia Breda-Stella, Cesar Alexandre Fabrega Carvalho

**Affiliations:** 1 Faculdade de Medicina de Jundiaí JundiaíSP Brazil Faculdade de Medicina de Jundiaí, Jundiaí, SP, Brazil.

**Keywords:** Tobacco smoke pollution, Kidney cortex, Kidney glomerulus, Mesangial cells, Diabetic nephropathies, Glomerular filtration rate, Rats, Wistar

## Abstract

**Objective:**

To analyze whether passive inhalation of cigarette smoke causes morphological, structural, and functional changes in kidneys of rats.

**Methods:**

Wistar rats, aged eight weeks, weighing on average 260g, were divided into Control Group and Smoking Group. Each group was subdivided into four groups of ten animals for morphofunctional analysis, in a period of seven and 28 days. The Smoking Group was exposed to smoke of 40 cigarettes per day, at certain times and in automated equipment for cigarette burning, called smoking machine (SM-MC-01). After the exposure period, urine and blood samples were collected for the functional analyses, and the kidneys were dissected and submitted to histological procedures for morphoquantitative analyses.

**Results:**

After exposure of animals of the Smoking Group, the following were observed: lower weight gain; lower water and feed intake; decreased renal weight, diameter, and volume; reduction in cortical thickness and glomerular volume density; decrease in glomerular and capsular diameter; increase in mesangial density; decreased urine volume; increased levels of glucose, serum creatinine and microalbuminuria; decreased urinary creatinine levels and creatinine clearance rate.

**Conclusion:**

Passive smoking negatively influences renal morphology and glomerular filtration rate, with effects similar to those described in the literature regarding active smoking.

## INTRODUCTION

According to the World Health Organization (WHO), smoking is the leading cause of preventable death in the world, affecting 8 million individuals per year, and approximately 1.2 million of these deaths result from individuals exposed to secondhand smoke.^(1)^ Cigarettes are composed of thousands of chemical components, recently estimated to be 5,600. Among them, 158 have toxicological properties capable of polluting the environment through smoke,^(2)^which, when inhaled by the passive smoker, contains six times more nicotine, four times more tar, seven times more carbon monoxide, and 73 times more ammonia than that inhaled by the active smoker.^(3)^

A comparative study performed by Dülger et al.,^(4)^ among a group of family members of smokers and volunteers who were not subjected to cigarette smoke, demonstrated renal functions are compromised by active smoking. However, Elihimas Júnior et al.,^(5)^ pointed out exposure to cigarette smoke can also be a potent kidney toxic agent. Jain et al.,^(6)^ have demonstrated that nicotine is a powerful stimulus for human mesangial cell proliferation and fibronectin production, aggravating glomerular injury.

Thus, smoking and circulating nicotine negatively influence the structures responsible for glomerular filtration, but the association between passive smoking and morphofunctional alterations of these structures, due to this exposure, has not been fully clarified in the literature yet, and this is the main objective of the present study.

## OBJECTIVE

To analyze the morphological, structural, and functional aspects in kidneys of rats after passive inhalation of cigarette smoke.

## METHODS

### Animal care

Animal care and housing were in accordance with the National and Institutional Guidelines for Animal Welfare, established by the *Colégio Brasileiro de Experimentação Animal* (COBEA) [Brazilian College of Animal Experimentation] and the *Conselho Nacional de Controle de Experimentação Animal* (CONCEA) [National Council for the Control of Animal Experimentation]. All use of animals was reviewed and approved by the Ethics Committee on Animal Experimentation (process 286/2015). Hence, efforts were made to minimize suffering, discomfort, and number of animals used, treating them humanely.

### Study design

An experimental study composed of male rats (*Rattus norvegicus albinus*), 8-week-old, weighing an average of 260g, divided into Control Group (CG) and Passive Smoker Group (SG). The CG and SG were subdivided into CG 7 days (n=10), SG 7 days (n=10), CG 28 days (n=10), and GT 28 days (n=10). The animals of both groups were kept in the vivarium of the *Faculdade de Medicina de Jundiaí* (FMJ), from November 2015 to January 2016, in separate rooms, two per box, under room temperature between 22±2°C, and a light/dark cycle of 12 hours. The animals’ weight gain (g), and intake of filtered water (mL), and feed (g) *ad libitum* (Nuvilab^®^; energy value: 339kcal=1418kJ 100.0%; carbohydrates 54g or 63.4%; proteins 22g or 25.9%; and lipids 4g or 10.6% - amounts per 100g serving and percentage of total energy value) were monitored throughout the experiment. The differences between the amount offered and the amount neglected by the animal were used to calculate the average intake.

**Figure 1:**
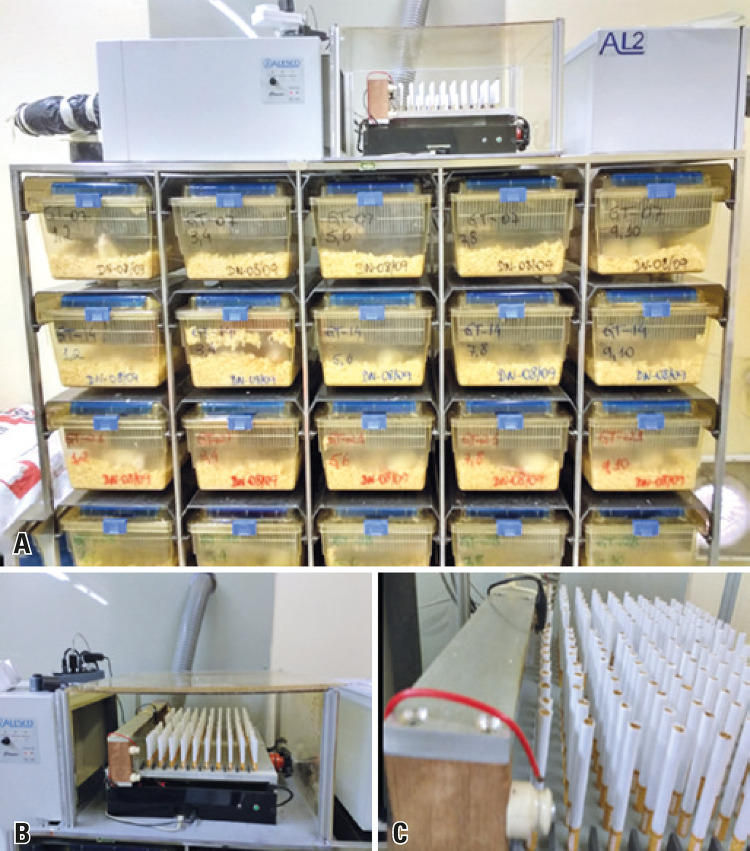
Photograph of the ventilation inlet system and the smoking machine. (A) Panoramic photograph of the ventilation support inlet system (smoking room). At top left is the blower in the center of the smoking machine, and at right is the exhaust fan. See also the back of the ducts and, in front, the sealed boxes attached to the system; (B) The smoking machine filled with 200 cigarettes, and, on the left, the inlet of the inflow system; (C) Detail of the smoking machine with the resistance system for automatic burning of cigarettes

To adapt to cigarette smoke, the animals of the SG were gradually exposed to burning cigarettes, which occurred in twos, up to 40 cigarettes per day (high yield; tar: 10mg; nicotine: 0.8mg; carbon monoxide: 10mg) for periods ranging from seven to 28 days. The burning occurred in an automated smoking machine (SM-MC-01), programmed to start burning cigarettes every 6 hours, with the burning of 10 cigarettes per cycle. The cigarette smoke was aspirated by the inflow system of the ventilation rack, the so-called “smoking room” (Figure 1), and distributed homogeneously inside the sealed boxes of the animals. Carbon dioxide was measured inside the boxes and in the environment where the CG and SG animals were kept, using a calibrated carbon dioxide meter (AZ Instruments, model AZ 77535, serial number 10109975) (certificate 00948/2016).

### Urine volume and analysis of the biochemical parameters

On the 6^th^ and 27^th^ days, the animals were placed in metabolic cages for collection of 24-hour urine. Soon after, they were weighed, anesthetized, euthanized by prolonged anesthesia, and the kidneys and blood were collected. Microalbuminuria was measured by immunoturbidimetry, through spectrophotometric analysis. The measurement of plasma creatinine clearance (reference value: 0.58±0.24mg/dL)^(7)^ and urinary creatinine clearance (reference value: 67.2±12.8mg/dL)^(7)^ was performed by the Jaffé method.^(8)^ For creatinine and glucose analyses (reference value: 108±17.4mg/dL),^(7)^ samples were centrifuged at 3,000rpm, for ten minutes, at 4°C to obtain serum. Enzymatic kits were then used for the measurements at Laborlab for creatinine, Bioclin for glucose, and Analisa for microalbuminuria.

### Weights, dimensions, and histology of kidneys

The kidneys obtained from the animals were dissected and weighed on an analytical scale (model AB204, Mettler Toledo, Barueri, SP, Brazil). The kidney dimensions (length, width, and height) were obtained using a pachymeter, and their radii (r) were used to determine the kidney volume in cubic centimeters using the formula vol=4/3π (r1.r2.r3), used to calculate the volume of an ellipse.^(9)^ For histological analysis, the right kidney (RK) was cut horizontally between the renal poles, and the left kidney (LK) had cross-section, cranial, and caudal to the kidney hilum sections, with the central section used for the analyses. The tissue sections were embedded in paraffin, cut approximately 4μm thick, and stained with hematoxylin-eosin (HE) and periodic acid Schiff (PAS).

### Morphometry and stereology

The measurements of the renal cortex were obtained with a calibrated millimeter eyepiece, attached to a Motic optical microscope with 4X objective lens, on histological slides of RK and LK, stained in HE.

**Figure 2:**
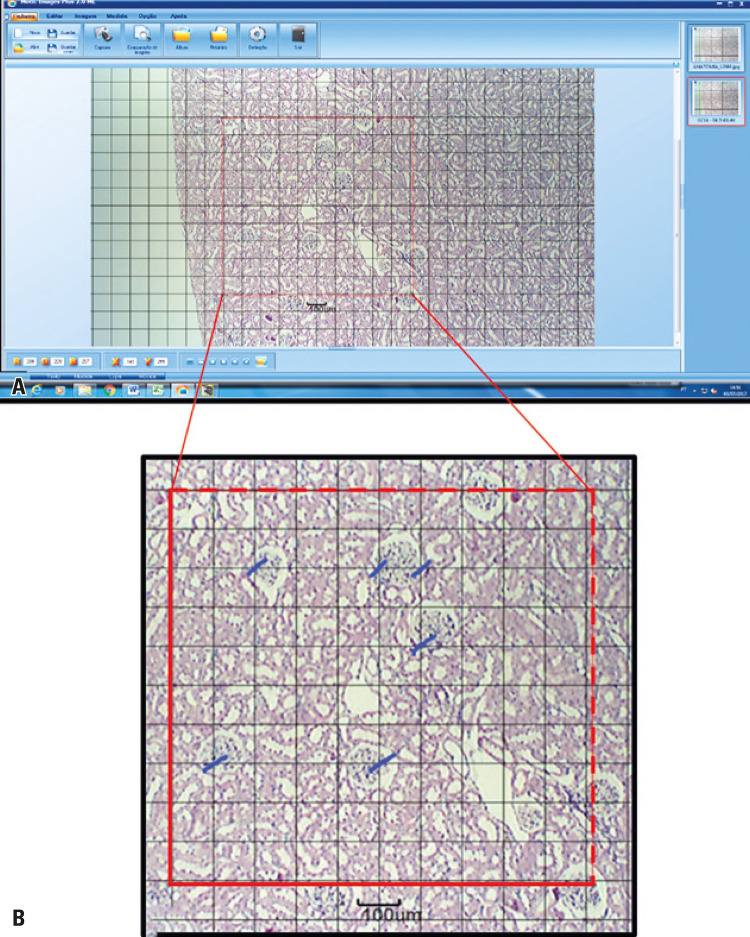
Schematic photomicrographs of the glomerular density measurement. (A) Schematic representation of the test system; (B) Enlarged photomicrograph of the schematic representation of the calibration of the points affecting the test system. Blue dashes represent points on the glomeruli, and dashed lines on the test system represent points prohibited for counting. 4X hematoxylin-eosin staining. Control Group 14, right kidney

The entire morphoquantitative study of the tissues at the structural level was performed using the Motic Images Plus 2.0 software, for the analyses and digitalization of the histological sections. To quantify the glomerular density of the groups, images of the renal cortex were captured with a 4X objective, on histological slides stained in HE. On the image, in 15 fields of the LK and 20 fields of the RK, a test system was inserted, formed by the reticule and the referred intersections, which totaled 100 points, counting those that focused on the glomeruli, and excluding those focusing on the dashed lines (Figure 2). The glomerular density in relation to the test system was calculated as follows: Vv[glom] =p/P (Vv[glom]), with glomerular density in percentage; p are the points on the glomeruli, and P is the total number of points in the test system.^(10)^

**Figure 3:**
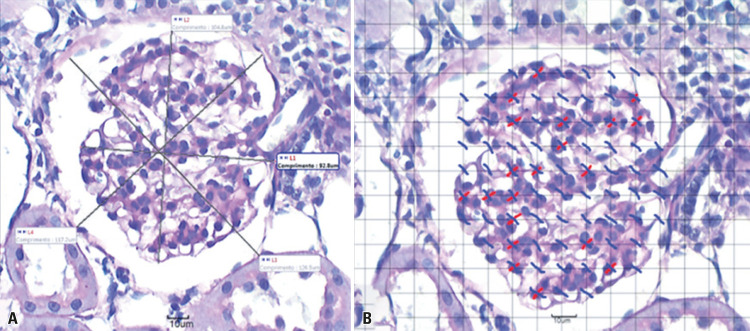
Schematic photomicrographs of the measurement of renal corpuscle diameters and mesangial density. (A) Enlarged photomicrograph of the glomerulus, showing at L1 and L2 the glomerular diameter measurements, and at L3 and L4 the glomerular capsule diameter measurements. Staining was done with Schiff’s periodic acid staining, 40X, Smoker Group 21, left kidney; (B) Schematic photomicrograph of the mesangial density measurement. Blue traces represent dots that are concentrated in the glomeruli, and red traces represent dots in the nucleus of mesangial cells. Schiff’s periodic acid staining, 60X, Smoker Group 21, left kidney

To calculate the glomerular and capsular diameters on the images captured with 40X objective on the slides stained with PAS, lines were drawn between the ends of these structures with the value calculated by the program (in µm). The mean diameter was obtained after averaging the largest and smallest diameter of the structures mentioned, in 40 renal corpuscles in the RK and 30 in the LK, in which vascular and/or urinary poles were observed (Figure 3A). The capsular spaces were obtained by the difference between their values.

In these same glomeruli, now with a 60X objective, the test system was inserted again, and the points that appeared on the glomerulus were counted. Immediately after, the points that were incident on the nuclei of the mesangial cell were counted;^(9)^ those were grouped within a mesangial space, surrounded by mesangial matrix between the glomerular capillary loops^(11)^ (Figure 3B). The volume fractions occupied by mesangial cell relative to the test system (glomerulus) were calculated as follows: Vv[mesg] = p/P (Vv[mesg], with mesangial volume density given as a percentage, p indicative of the points on the mesangial cell nuclei, and P corresponding to the total number of points in the test system.^(8)^

### Statistical analysis

The statistical analysis was carried out with the objective of making comparisons between the CG and the SG, as well as intra-group comparisons, for each of the variables studied, using means and standard deviations. The data were submitted to analysis of variance (ANOVA). The assumption of homogeneity of variances was verified by applying Tukey’s multiple comparisons test. The significance level adopted for all tests was p≤0.05. The software used for analysis was Biostat 5.3 (*Instituto de Desenvolvimento Sustentável Mamirauá,* Belém, PA, Brazil), and comparisons were made, both between CG and SG, and intra-group.

## RESULTS

### Carbon dioxide levels (part per million)

The efficacy of the smoking room equipment in generating and insufflating smoke was confirmed by detection of carbon dioxide levels. The initial mean value before cigarette burning was 620.8 parts per million (ppm) in the SG environment, which was close to the mean value obtained in the CG environment (571.8ppm). As the cigarettes were lit and smoke insufflated into the boxes, carbon dioxide levels increased progressively from a minimum mean value of 616.6ppm to a maximum mean value of 1,267.0ppm (p≤0.01).

### Body weight gain and intake

The SG animals showed lower body weight gain, and lower feed and water intake during the periods studied (Table 1).

**Table 1:** Body weight gain and intake

Variables	Groups			

	7 days		28 days	
	
	CG	SG	CG	SG
Weight, g	289.1±18.4	265.0±17.2*	377.6±32.7	344.2±29.0^†^
Feed, g	194.5±1.5	103.0±6.9*	199.5±14.4	148.3±11.8*
Water, mL	288.0±28.4	178.0±17.8*	286.0±24.3	252.3±13.5*

Results expressed as mean±standard deviation of the analyses inter- and intra-groups.

* Differed by 1% significance (p≤0.01); ^†^ differed by 5% significance (p≤0.05).

CG: Control Group; SG: Smoker Group.

### Morphometry: kidney weights and dimensions

There were significant reductions in the mean values for kidney weight, kidney dimensions (length, width, and height), kidney volume, and cortical thickness in the SG animals during the periods studied. There was no difference as to the length of the RK in both periods, and the height of the LK in the seven day period (Table 2).

**Table 2:** Kidney weight and dimensions at seven and 28 days

Variables	Groups			

	Control		Smoker	
	
	RK	LK	RK	LK
Kidney weight, g				
7 days	1.51±0.06	1.45±0.03	1.28±0.16*	1.23±0.17^†^
28 days	1.79±0.06	1.68±0.16	1.41±0.21^†^	1.34±0.18^†^
Length, mm				
7 days	18.8±0.45	20.7±0.57	18.5±1.02	18.7±0.56^†^
28 days	19.8±0.26	20.7±0.47	19.7±0.93	19.7±1.09*
Width, mm				
7 days	12.3±0.48	11.8±0.27	10.5±0.51^†^	9.7±0.29^†^
28 days	12.8±0.29	12.4±0.33	11.7±0.82^†^	11.1±0.36^†^
Height, mm				
7 days	9.6±0.61	8.8±0.07	8.2±0.65*	8.6±0.11
28 days	9.5±0.61	9.4±0.40	8.6±0.91*	8.7±0.52*
Kidney volume, cm^3^				
7 days	1.15±0.08	1.13±0.07	0.84±0.11^†^	0.82±0.11^†^
28 days	1.27±0.08	1.27±0.05	1.04±0.18^†^	1.00±0.12^†^
Cortical thickness, mm				
7 days	2.31±0.12	2.19±0.08	2.00±0.10^†^	1.93±0.07^†^
28 days	2.36±0.13	2.26±0.10	2.10±0.15^†^	2.13±0.03^†^

Results expressed as mean±standard deviation of the analyses inter- and intra-groups.

* Differed by 5% significance (p≤0.05); ^†^ differed by 1% significance (p≤0.01).

RK: right kidney; LK: left kidney.

### Morphometry and stereology of renal corpuscles

**Figure 4:**
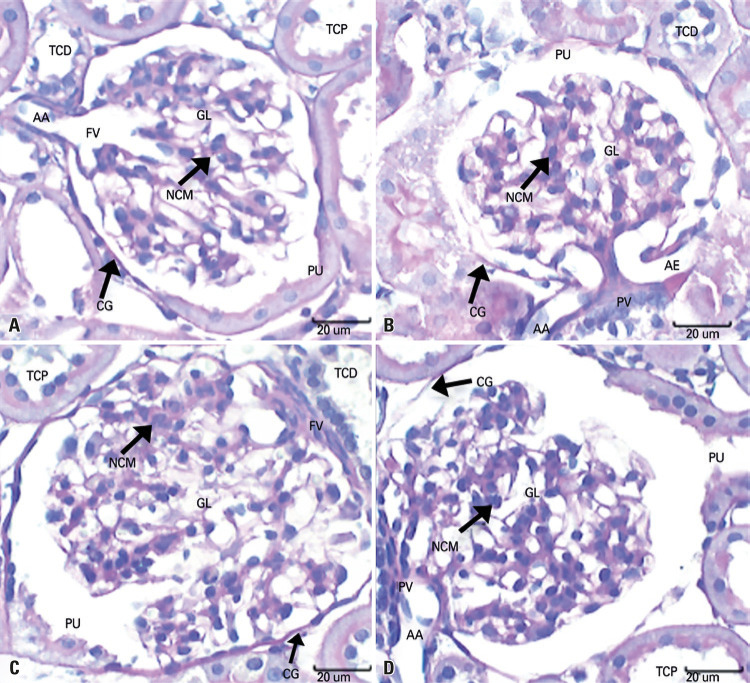
Photomicrographs of the cortical region of the kidneys at seven and 28 days. (A and C) Control Group; (B and D) Smoker Group. Note that, in B and D, there are smaller glomeruli with mesangial cells in higher concentration, when compared to A and C. Note glomerular capsule, distal convoluted tubule, proximal convoluted tubule, urinary pole, vascular pole, afferent arteriole, and mesangial cell nucleus

TCD: distal convoluted tubule; GL: glomeruli; TCP: proximal convoluted tubule; PU: urinary pole; CG: glomerular capsule; NCM: mesangial cell nucleus; PV: vascular pole; AA: afferent arteriole; AE: efferent arteriole.

As for the glomerular volume density and the glomerular and capsular diameters, the mean values observed in the SG kidneys were significantly lower. Regarding capsular spaces, the mean values observed in the SG were significantly higher during the period of 28 days. Regarding the density of mesangial volume occupied by mesangial cell in glomeruli, the mean values in the SG were significantly higher (Table 3 and Figure 4).

**Table 3:** Glomerular volume density, mesangial volume density, and glomerular corpuscle diameter at seven and 28 days

Variables	Groups			

	Control		Smoker	
	
	RK	LK	RK	LK
Glomerular volume density				
7 days	4.8±0.18	4.8±0.20	3.7±0.30*	4.0±0.18*
28 days	5.1±0.22	5.1±0.21	4.2±0.24*	4.3±0.17*
Mesangial volume density				
7 days	16.2±1.4	12.1±1.2	18.8±1.9^†^	18.2±2.1*
28 days	11.2±0.7	11.3±0.4	15.1±1.3*	14.2±1.6*
Glomerulus, µm				
7 days	88.6±1.8	86.9±2.7	79.8±1.8*	81.1±3.3^†^
28 days	104.6±4.6	100.7±7.3	88.6±4.6*	86.7±1.8*
Glomerulus, µm				
7 days	103.2±3.4	102.0±2.8	95.6±3.2*	96.7±3.9^†^
28 days	115.5±6.4	112.5±7.0	105.3±5.9^†^	104.4±2.5^†^
Glomerular capsule, µm				
7 days	14.6±2.5	15.1±1.4	16.7±2.0	15.6±1.0
28 days	10.9±3.1	11.8±1.6	16.7±2.5^†^	17.7±1.8^†^

Results expressed as mean±standard deviation of the analyses inter- and intra-groups.

* Differed by 1% significance (p≤0.01); ^†^ differed by 5% significance (p≤0.05).

RK: right kidney; LK: left kidney.

### Urine volume and analysis of biochemical parameters

The urinary volume of the SG animals was significantly lower at seven days, and there was no difference at 28 days. The glucose, creatinine, and microalbuminuria levels of the SG animals were significantly higher, and the urinary creatinine levels and creatinine clearance rate in the SG animals were significantly lower during the periods studied (Table 4).

**Table 4:** Urine volume and analysis of biochemical parameters

Variables	Groups			

	7 days		28 days	
	
	CG	SG	CG	SG
Urine volume, mL	14.5±8.0	10.6±10.0*	10.7±6.7	11.6±9.2
Glucose, mg/dL	93.8±17.0	133.6±32.2*	96.2±22.3	118.4±17.5^†^
Creatinine, mg/dL	0.61±0.04	0.72±0.15^†^	0.68±0.07	0.84±0.06*
Urinary creatinine, mg/dL	110.7±62.3	55.9±29.9^†^	181.0±69.1	115.9±66.6^†^
Creatinine clearance, mL/min	1.43±0.60	0.45±0.40*	1.65±0.54	0.75±0.30*
Microalbum, mg/L	0.47±0.23	6.37±2.2*	0.41±0.20	4.45±1.50*

Results expressed as mean±standard deviation of the analyses inter- and intra-groups.

* Differed by 1% significance (p≤0.01); ^†^ differed by 5% significance (p≤0.05).

CG: Control Group; SG: Smoker Group.

## DISCUSSION

In the present study, carbon dioxide was used to verify the degree of environmental pollution from cigarette smoke. The levels of this gas in the boxes of the SG animals were similar to those found by Colli Neto et al.,^(12)^ who observed levels of carbon dioxide concentration above 999ppm. This indicates the components present in cigarette smoke circulated and the animals inhaled them. Furthermore, the work of Robello^(13)^ using a similar method of exposure identified high levels of cotinine (nicotine metabolite) in the serum of animals from the SG.

Because of this exposure, the SG animals presented with lower weight gain in the periods studied, due to the lower intake of water and feed. Nicotine inhalation by cigarette smoke alters the metabolism, promoting increased heat production and oxygen consumption, which stimulates the thyroid-stimulating hormone (TSH) with consequent increased metabolism, and higher levels of glucose and cholesterol in the blood.^(14)^ It also causes an acute elevation of dopamine and serotonin concentrations in the brain, thus inhibiting food intake.^(15)^ One of the hypotheses suggested for this is the moderate hyperglycemic level in smokers,^(16)^ a fact observed in the present study and in the studies by Sinzato et al.^(17)^

In the study with rats exposed to smoking as from the intrauterine period, Schiffner^(18)^observed that analysis of the glucose tolerance test showed resistance to insulin action and higher glucose peaks in this group when compared to controls, suggesting that smokers have greater difficulty in glucose uptake due to the lower availability of insulin, the lower number and/or function of insulin receptors, or the lower capacity to induce the intracellular signaling cascade, which facilitates glucose uptake.

As a result of these factors, kidney weights and dimensions in the present study were affected, culminating in smaller kidney volumes, a fact also observed in the experimental study by Dündar et al.^(19)^ However, although certain morphological alterations may not mean impairment of renal function, this association seems to follow in a directly proportional manner in smoking, since significant atrophy was observed when assessing the cortical thickness in the SG. In fact, the relation between cortical thickness and kidney function has been suggested, especially when there is a proportional reduction of glomeruli,^(20)^ also observed in the present study, with a significant reduction of the dimensions of the renal corpuscle diameter. However, the capsular space increased in the SG, with longer exposure time, probably due to reductions in glomerular diameter, allowing the resulting decrease in glomerular filtration rate (GFR).^(19)^

In the evaluation of mesangial volume density, there was a significant increase in the SG animals. The mesangial cell that help and modulate the GFR and the surface area of the glomerulus, thanks to their ability to contract and relax in response to vasoactive drugs, may suffer significant proliferation, with glomerular sclerosis and tubulo-interstitial fibrosis in animals in response to the glomerular endothelial lesions caused by smoking.^(21,22)^

Due to the morphological changes observed, as well as a lower water consumption by the SG animals, also observed in the studies by Gonçalves-Silva et al.,^(23)^ the biochemical parameters of blood and urine analysis also changed in the SG, providing subsidies to understand the possible damage to kidney function, since the levels of creatinine, urinary creatinine, and urinary volume, used to determine the creatinine clearance rate to estimate the GFR, were significantly lower. Smoking promotes a significant increase in the albumin-creatinine ratio, with changes in glomerular capillary pressure and kidney function,^(24)^ and there are few studies correlating nicotine present in the bloodstream of passive smokers with kidney diseases, especially regarding acute exposure.^(25)^

Thus, the acute action of circulating nicotine may be related to glomerular endothelial dysfunction, since the persistent levels of microalbuminuria, significantly elevated in this study, determine and classify the onset and progression of nephropathy, and express a risk factor for endothelial dysfunction and injury in the macro- and microcirculation.^(26)^ Cooper^(27)^ demonstrated nicotine accelerates nephropathies, which are detected by increasing microalbuminuria with progression to proteinuria, concluding that active and passive smoking are toxic to kidney function.

## CONCLUSION

Exposure and passive inhalation of cigarette smoke by the animals caused morphological and structural changes in their kidneys. Coupled with changes in biochemical parameters due to exposure, the result was a decreased glomerular filtration rate, demonstrating exposure to secondhand smoke can compromise kidney function, with similar effects to those of active smoking.
