# Supplementary material for: Morphofunctional study on the effects of passive smoking in kidneys of rats
Source: Einstein (Sao Paulo). 2021 Oct 25;19:eAO6000. doi: 10.31744/einstein_journal/2021AO6000 (PMC8555876; doi:10.31744/einstein_journal/2021AO6000)
Supplement: Translation

# Translation: Estudo morfofuncional sobre os efeitos do tabagismo passivo em rins de ratos

**Authors:** Carlos Alberto de Moraes, Mercia Breda-Stella, Cesar Alexandre Fabrega Carvalho

**Affiliations:** 1 Brasil Faculdade de Medicina de Jundiaí, Jundiaí, SP, Brasil.

**Keywords:** Poluição por fumaça de tabaco, Córtex renal, Glomérulos renais, Células mesangiais, Nefropatias diabéticas, Taxa de filtração glomerular, Ratos Wistar

## Abstract

**Objetivo:**

Analisar se a inalação passiva da fumaça do cigarro proporciona alterações morfológicas, estruturais e funcionais nos rins de ratos.

**Métodos:**

Ratos Wistar, com oito semanas de idade, pesando, em média, 260g, foram divididos em Grupo Controle e Grupo Tabagista. Cada grupo foi subdividido em quatro grupos de dez animais para análise morfofuncional, em um período de sete e 28 dias. O Grupo Tabagista foi exposto à fumaça de 40 cigarros por dia, em horários determinados e equipamento automatizado de queima de cigarros, denominado *smoking machine* (SM-MC-01). Após o período de exposição, foram coletadas amostras de urina e sangue para as análises funcionais, e os rins foram dissecados e submetidos a procedimentos histológicos para análises morfoquantitativas.

**Resultados:**

Após a exposição dos animais do Grupo Tabagista, observou-se menor ganho de peso; menor consumo de água e ração; menor peso, diâmetro e volume renal; redução em espessura cortical e densidade de volume glomerular; diminuição no diâmetro glomerular e capsular; aumento na densidade mesangial; volume urinário diminuído; níveis aumentados de glicose, creatinina sérica e microalbuminúria; níveis reduzidos de creatinina urinária e redução da taxa de depuração da creatinina.

**Conclusão:**

O tabagismo passivo influencia negativamente na morfologia renal e na taxa de filtração glomerular, com efeitos semelhantes aos descritos na literatura em relação ao tabagismo ativo.

## INTRODUÇÃO

Segundo a Organização Mundial da Saúde (OMS), o tabagismo é a principal causa de morte evitável no mundo, acometendo 8 milhões de indivíduos por ano, e, aproximadamente 1,2 milhões de mortes são de indivíduos expostos ao tabagismo passivo.^(1)^ O cigarro é composto de milhares de componentes químicos, recentemente estimados em 5.600. Entre eles, 158 têm propriedades toxicológicas capazes de poluir o meio ambiente por meio da fumaça,^(2)^ a qual, quando inalada pelo tabagista passivo, contém seis vezes mais nicotina, quatro vezes mais alcatrão, sete vezes mais monóxido de carbono e 73 vezes mais amônia que a tragada pelo fumante ativo.^(3)^

Estudo comparativo realizado por Dülger et al.,^(4)^ entre um grupo de familiares de pessoas fumantes e voluntários que não estavam sujeitos à fumaça do cigarro, demonstrou que os rins têm as funções comprometidas mediante o tabagismo ativo. Porém, Elihimas Júnior et al.,^(5)^ apontam que a exposição à fumaça do cigarro pode ser também um potente agente tóxico renal. Jain et al.,^(6)^ demonstraram que a nicotina é um poderoso estímulo para a proliferação de células mesangiais humanas e produção de fibronectina, agravando a lesão glomerular.

Desta forma, o tabagismo e a nicotina circulante influenciam negativamente nas estruturas responsáveis pela filtração glomerular, mas a associação entre o tabagismo passivo e as alterações morfofuncionais dessas estruturas, diante dessa exposição, não está totalmente esclarecida na literatura, sendo este o objetivo central do presente trabalho.

## OBJETIVO

Analisar os aspectos morfológicos, estruturais, e funcionais nos rins de ratos após inalação passiva da fumaça do cigarro.

## MÉTODOS

### Cuidado dos animais

O cuidado e o alojamento dos animais estavam de acordo com as Diretrizes Nacionais e Institucionais para o Bem-Estar Animal, estabelecidas pelo Colégio Brasileiro de Experimentação Animal (COBEA) e Conselho Nacional de Controle de Experimentação Animal (CONCEA). Todo o uso de animais foi analisado e aprovado pelo Comitê de Ética em Experimentação Animal (processo 286/2015). Assim, tiveram-se os esforços para minimizar o sofrimento, o desconforto e o número de animais utilizados, tratando-os com humanidade.

### Desenho do estudo

Estudo experimental composto por ratos machos (*Rattus norvegicus albinus*), com oito semanas de idade, pesando uma média de 260g, divididos em Grupo Controle (GC) e Grupo de Tabagista passivo (GT). O GC e o GT foram subdivididos em: GC 7 dias (n=10), GT 7 dias (n=10), GC 28 dias (n=10) e GT 28 dias (n=10). Os animais de ambos os grupos foram mantidos no biotério da Faculdade de Medicina de Jundiaí (FMJ), no período de novembro de 2015 a janeiro de 2016, em salas separadas, com a quantidade de dois por caixa, com temperatura ambiente entre 22±2°C e ciclo claro/escuro de 12 horas. O ganho de peso dos animais (g) e a ingestão de água filtrada (mL) e ração (g) *ad libitum* (Nuvilab^®^; valor energético: 339kcal=1418kJ 100,0%; carboidratos 54g ou 63,4%; proteínas 22g ou 25,9% e lipídios 4g ou 10,6% – quantidades por porção de 100g e percentagem do valor calórico total) foram monitorados ao longo do experimento, sendo que as diferenças entre a quantidade oferecida e a negligenciada pelo animal foram utilizadas para calcular o consumo médio.

Para a adaptação à fumaça do cigarro, os animais do GT foram expostos gradativamente à queima deles, que ocorreu de dois em dois, até completar o total de 40 cigarros diários (alto teor; alcatrão: 10mg; nicotina: 0,8mg; monóxido de carbono: 10mg) por períodos variados de sete e 28 dias. A queima ocorreu no equipamento automatizado, denominado *smoking machine* (SM-MC-01), programado para iniciar a queima dos cigarros a cada 6 horas, com o acendimento de dez a cada ciclo. As fumaças dos cigarros foram aspiradas pelo sistema de influxo da estante de ventilação, o chamado “fumódromo” (Figura 1), e distribuídas de modo homogêneo para o interior das caixas seladas dos animais. O dióxido de carbono foi medido no interior das caixas e no ambiente onde ficavam os animais do GC e GT, por meio de um medidor de dióxido de carbono (AZ Instruments, modelo AZ 77535, número de série 10109975) calibrado (certificado 00948/2016).

**Figura 1:** Fotografia do sistema de entrada de ventilação e da *smoking machine.* (A) Fotografia panorâmica do sistema de entrada do suporte de ventilação (fumódromo). Na parte superior esquerda, o insuflador, no centro da *smoking machine*, e, à direita, o exaustor. Veja ainda a parte traseira dos dutos e, na frente, as caixas seladas presas ao sistema; (B) *Smoking machine* abastecida com 200 cigarros, estando, à esquerda, a entrada do sistema de influxo; (C) Detalhe da *smoking machine* com o sistema de resistências para queima automática de cigarros

### Volume de urina e análise dos parâmetros bioquímicos

No 6º e no 27º dias, os animais foram colocados em gaiola metabólica para a coleta da urina de 24 horas. Logo após, foram pesados, anestesiados e submetidos à eutanásia por prolongamento anestésico, sendo coletados os rins e o sangue. A mensuração da microalbuminúria foi realizada por imunoturbidimetria, por meio de análise em espectrofotometria. A mensuração da depuração da creatinina plasmática (valor de referência: 0,58±0,24mg/dL)^(7)^ e urinária (valor de referência: 67,2±12,8mg/dL)^(7)^ foi realizada pelo método de Jaffé.^(8)^ Para as análises de creatinina e glicose (valor de referência: 108±17,4mg/dL),^(7)^ as amostras foram centrifugadas a 3.000rpm, durante dez minutos, a 4°C, para a obtenção do soro. Foram, então, utilizados *kits* enzimáticos para as mensurações da Laborlab para creatinina, Bioclin para glicose e da Analisa para microalbuminúria.

### Pesos, dimensões e histologia dos rins

Os rins obtidos dos animais foram dissecados e pesados em balança analítica (modelo AB204, Mettler Toledo, Barueri, SP, Brasil). As dimensões renais (comprimento, largura e altura) foram obtidas por meio de um paquímetro, e seus raios (r) foram utilizados para determinar o volume renal em centímetros cúbicos por meio da fórmula vol=4/3π (r1.r2.r3), usada para calcular o volume de uma elipse.^(9)^ Para análise histológica, o rim direito (RD) foi submetido a um corte horizontal entre os polos renais e o rim esquerdo (RE) a duas seções transversais, cranial e caudal ao hilo renal, sendo a central utilizada para as análises. As secções de tecido foram embebidas em parafina, cortadas com aproximadamente 4 μm de espessura e coradas em hematoxilina-eosina (HE) e ácido periódico de Schiff (PAS - *periodic acid Schiff*).

### Morfometria e estereologia

As medidas do córtex renal foram obtidas com uma ocular milimetrada, calibrada, acoplada ao microscópio óptico Motic com objetiva de 4X, em lâminas histológicas do RD e RE, coradas em HE.

Todo o estudo morfoquantitativo dos tecidos em nível estrutural foi realizado por meio do programa Motic Images Plus 2.0, para as análises e a digitalização dos cortes histológicos. Para a quantificação da densidade glomerular dos grupos, foram capturadas imagens do córtex renal com objetiva de 4X, em lâminas histológicas coradas em HE. Sobre a imagem, em 15 campos do RE e 20 campos do RD, foi inserido um sistema de teste, formado pelo retículo e pelas referidas intersecções, que somaram 100 pontos, contabilizando os que incidiam sobre os glomérulos, excetuando-se aqueles que incidiam sobre as linhas tracejadas (Figura 2). A densidade glomerular em relação ao sistema de teste foi calculada da seguinte forma: Vv[glom] = p/P (Vv[glom]), com densidade glomerular em percentagem, p são os pontos sobre os glomérulos, e P é o número total de pontos do sistema de teste.^(10)^

**Figura 2:** Fotomicrografias esquemáticas da medida da densidade glomerular. (A) Representação esquemática do sistema de teste; (B) Fotomicrografia ampliada da representação esquemática da calibração dos pontos que afetam o sistema de teste. Traços azuis representam pontos nos glomérulos, e linhas tracejadas no sistema de teste representam pontos proibidos para a contagem. Coloração por hematoxilina-eosina 4X. Grupo Controle 14, rim direito

Para o cálculo dos diâmetros glomerulares e capsulares, sobre as imagens capturadas com objetiva de 40X nas lâminas coradas em PAS, foram traçadas linhas entre as extremidades dessas estruturas com o valor calculado pelo programa (em µm). O diâmetro médio foi obtido após a média do maior e menor diâmetro das estruturas mencionadas, em 40 corpúsculos renais no RD e 30 no RE, em que fossem observados os polos vasculares e/ou urinários (Figura 3A). Os espaços capsulares foram obtidos por meio da diferença entre seus valores encontrados.

**Figura 3:** Fotomicrografias esquemáticas da medida dos diâmetros do corpúsculo renal e da densidade mesangial. (A) Fotomicrografia ampliada do glomérulo, demonstrando, em L1 e L2, as medidas do diâmetro glomerular e, em L3 e L4, as medidas do diâmetro da cápsula glomerular. Coloração por ácido periódico de Schiff, 40X, Grupo Tabagista 21, rim esquerdo; (B) Fotomicrografia esquemática da medida da densidade mesangial. Traços azuis representam pontos que se concentram nos glomérulos, e traços vermelhos representam pontos no núcleo das células mesangiais. Coloração por ácido periódico de Schiff, 60X, Grupo Tabagista 21, rim esquerdo

Nestes mesmos glomérulos, agora com objetiva de 60X, foi inserido novamente o sistema de teste, e foram contabilizados os pontos que incidiam no glomérulo. Logo após, foram contabilizados os pontos que incidiam sobre os núcleos das células mesangiais,^(9)^ sendo aqueles agrupados dentro de um espaço mesangial, envoltos por matriz mesangial entre as alças dos capilares glomerulares^(11)^(Figura 3B). As frações de volume ocupadas pelas células mesangiais, em relação ao sistema de teste (glomérulo) foram calculadas da seguinte forma: Vv[mesg] = p/P (Vv[mesg], sendo a densidade de volume mesangial dada em percentagem, p indicativo dos pontos sobre os núcleos das células mesangiais, e P correspondente ao número total de pontos do sistema de teste.^(8)^

### Análise estatística

A análise estatística foi realizada com o objetivo de realizar comparações tanto entre o GC e o GT, quanto como intragrupos, em cada uma de suas variáveis, sendo estas estudadas por meio de médias e desvios-padrão. Os dados foram submetidos à análise de variância (ANOVA). A suposição de homogeneidade das variâncias foi verificada aplicando-se os testes de comparações múltiplas pelo método de Tukey. O nível de significância assumido para todos os testes empregados foi p≤0,05. O software utilizado para análise foi o Biostat 5.3 (Instituto de Desenvolvimento Sustentável Mamirauá, Belém, PA, Brasil), e foram realizadas comparações, tanto entre GC e GT, como intragrupo.

## RESULTADOS

### Níveis de dióxido de carbono (parte por milhão)

A eficácia do equipamento fumódromo na geração e na insuflação de fumaça foi confirmada pela detecção dos níveis de dióxido de carbono. O valor médio inicial antes da queima dos cigarros foi de 620,8 parte por milhão (ppm) no ambiente do GT, sendo este próximo ao valor médio obtido no ambiente do GC (571,8ppm). Com o acendimento dos cigarros e a insuflação da fumaça para as caixas, os níveis de dióxido de carbono aumentaram progressivamente a partir de um valor médio mínimo de 616,6 ppm, até o valor médio máximo de 1.267,0ppm (p≤0,01).

### Ganho de peso corporal e consumos

Os animais do GT apresentaram menor ganho de peso corporal e menor consumo de ração e água nos períodos estudados (Tabela 1).

**Tabela 1:** Ganho de peso corporal e consumos

Variáveis	Grupos			

	7 dias		28 dias	
	
	GC	GT	GC	GT
Peso, g	289,1±18,4	265,0±17,2*	377,6±32,7	344,2±29,0^†^
Ração, g	194,5±1,5	103,0±6,9*	199,5±14,4	148,3±11,8*
Água, mL	288,0±28,4	178,0±17,8*	286,0±24,3	252,3±13,5*

Resultados expressos por média±desvio padrão das análises entre e intragrupos.

* Diferem em 1% de significância (p≤0,01); ^†^ diferem em 5% de significância (p≤0,05).

GC: Grupo Controle; GT: Grupo Tabagista.

### Morfometria: pesos e dimensões renais

Foram observadas reduções significativas nos valores médios referentes a peso dos rins; dimensões renais (comprimento, largura e altura); volume renal e a espessura cortical nos animais do GT nos períodos estudados. Não houve diferença quanto ao comprimento no RD em ambos os períodos e quanto à altura do RE no período de sete dias (Tabela 2).

**Tabela 2:** Peso e dimensões renais no período de sete e 28 dias

Variáveis	Grupos			

	Controle		Tabagista	
	
	RD	RE	RD	RE
Peso rim, g				
7 dias	1,51±0,06	1,45±0,03	1,28±0,16*	1,23±0,17^†^
28 dias	1,79±0,06	1,68±0,16	1,41±0,21^†^	1,34±0,18^†^
Comprimento, mm				
7 dias	18,8±0,45	20,7±0,57	18,5±1,02	18,7±0,56^†^
28 dias	19,8±0,26	20,7±0,47	19,7±0,93	19,7±1,09*
Largura, mm				
7 dias	12,3±0,48	11,8±0,27	10,5±0,51^†^	9,7±0,29^†^
28 dias	12,8±0,29	12,4±0,33	11,7±0,82^†^	11,1±0,36^†^
Altura, mm				
7 dias	9,6±0,61	8,8±0,07	8,2±0,65*	8,6±0,11
28 dias	9,5±0,61	9,4±0,40	8,6±0,91*	8,7±0,52*
Volume renal, cm^3^				
7 dias	1,15±0,08	1,13±0,07	0,84±0,11^†^	0,82±0,11^†^
28 dias	1,27±0,08	1,27±0,05	1,04±0,18^†^	1,00±0,12^†^
Espessura cortical, mm				
7 dias	2,31±0,12	2,19±0,08	2,00±0,10^†^	1,93±0,07^†^
28 dias	2,36±0,13	2,26±0,10	2,10±0,15^†^	2,13±0,03^†^

Resultados expressos por média±desvio padrão das análises entre e intragrupos.

* Diferem em 5% de significância (p≤0,05); ^†^ diferem em 1% de significância (p≤0,01).

RD: rim direito; RE: rim esquerdo.

### Morfometria e estereologia dos corpúsculos renais

Quanto à densidade do volume glomerular e aos diâmetros glomerulares e capsulares, os valores médios observados nos rins do GT foram significativamente menores. Referente aos espaços capsulares, os valores médios observados no GT foram significativamente maiores no período de 28 dias. Já em relação à densidade do volume mesangial, ocupada pelas células mesangiais nos glomérulos, os valores médios no GT foram significativamente maiores (Tabela 3 e Figura 4).

**Tabela 3:** Densidade do volume glomerular, densidade do volume mesangial e diâmetro do corpúsculo glomerular no período de sete e 28 dias

Variáveis	Grupos			

	Controle		Tabagista	
	
	RD	RE	RD	RE
Densidade do volume glomerular				
7 dias	4,8±0,18	4,8±0,20	3,7±0,30*	4,0±0,18*
28 dias	5,1±0,22	5,1±0,21	4,2±0,24*	4,3±0,17*
Densidade do volume mesangial				
7 dias	16,2±1,4	12,1±1,2	18,8±1,9^†^	18,2±2,1*
28 dias	11,2±0,7	11,3±0,4	15,1±1,3*	14,2±1,6*
Glomérulo, µm				
7 dias	88,6±1,8	86,9±2,7	79.8±1,8*	81,1±3,3^†^
28 dias	104,6±4,6	100,7±7,3	88,6±4,6*	86,7±1,8*
Cápsula glomerular, µm				
7 dias	103,2±3,4	102,0±2,8	95,6±3,2*	96,7±3,9^†^
28 dias	115,5±6,4	112,5±7,0	105,3±5,9^†^	104,4±2,5^†^
Espaço capsular				
7 dias	14,6±2,5	15,1±1,4	16,7±2,0	15,6±1,0
28 dias	10,9±3,1	11,8±1,6	16,7±2,5^†^	17,7±1,8^†^

Resultados expressos por média±desvio padrão das análises entre e intragrupos.

* Diferem em 1% de significância (p≤0,01); ^†^ diferem em 5% de significância (p≤0,05).

RD: rim direito; RE: rim esquerdo.

**Figura 4:** Fotomicrografias da região cortical dos rins com sete e 28 dias. (A e C) Grupo Controle; (B e D) Grupo Tabagista. Observar, em B e D, glomérulos menores com células mesangiais em maior concentração, quando comparados a A e C

TCD: túbulo contorcido distal; GL: glomérulos; TCP: túbulo contornado proximal; PU: polo urinário; CG: cápsula glomerular; NCM: núcleo da célula mesangial; PV: polo vascular; AA: arteríola aferente; AE: arteríola eferente.

### Volume de urina e análise dos parâmetros bioquímicos

O volume urinário dos animais do GT foi significativamente menor no período de sete dias e não houve diferença em 28 dias. Os níveis de glicose, creatinina e de microalbuminúria dos animais do GT foram significativamente maiores, e os níveis de creatinúria e a taxa de depuração de creatinina nos animais do GT foram significativamente menores nos períodos estudados (Tabela 4).

**Tabela 4:** Volume de urina e análise dos parâmetros bioquímicos

Variáveis	Grupos			

	7 dias		28 dias	
	
	GC	GT	GC	GT
Volume urinário, mL	14,5±8,0	10,6±10,0*	10,7±6,7	11,6±9,2
Glicose, mg/dL	93,8±17,0	133,6±32,2*	96,2±22,3	118,4±17,5^†^
Creatinina, mg/dL	0,61±0,04	0,72±0,15^†^	0,68±0,07	0,84±0,06*
Creatinúria, mg/dL	110,7±62,3	55,9±29,9^†^	181,0±69,1	115,9±66,6^†^
Depuração de creatinina, mL/min	1,43±0,60	0,45±0,40*	1,65±0,54	0,75±0,30*
Microalbum, mg/L	0,47±0,23	6,37±2,2*	0,41±0,20	4,45±1,50*

Resultados expressos por média±desvio padrão das análises entre e intragrupos.

* Diferem em 1% de significância (p≤0,01); ^†^ diferem em 5% de significância (p≤0,05).

GC: Grupo Controle; GT: Grupo Tabagista.

## DISCUSSÃO

No presente estudo, o dióxido de carbono foi utilizado para verificar o grau de poluição ambiental pela fumaça do cigarro. Os níveis deste gás nas caixas dos animais do GT foram semelhantes aos encontrados em Colli Neto et al.,^(12)^que observaram níveis de concentração de dióxido de carbono acima de 999ppm. Isso indica que os componentes presentes na fumaça do cigarro circularam e foram inalados pelos animais. Além disso, o trabalho de Robello^(13)^ utilizando um método semelhante de exposição identificou altos níveis de cotinina (metabólito da nicotina) no soro de animais do GT.

Como resultado dessa exposição, os animais do GT apresentaram menor ganho de peso nos períodos estudados, devido à menor ingestão de água e ração, pois a inalação da nicotina pela fumaça do cigarro altera o metabolismo, promovendo aumento da produção de calor e no consumo de oxigênio, estimulação do hormônio tireoestimulante (TSH), com aumento do metabolismo, e níveis de glicose e colesterol aumentados no sangue,^(14)^ além de causar a elevação aguda da concentração de dopamina e serotonina no cérebro, que inibem a ingestão de alimentos.^(15)^ Uma das hipóteses sugeridas para isso é o nível hiperglicêmico moderado nos tabagistas,^(16)^ fato observado no presente trabalho e nos estudos de Sinzato et al.^(17)^

Schiffner^(18)^ observou, em seu estudo com ratos expostos ao tabagismo desde o período intrauterino, que a análise do teste de tolerância à glicose mostrou, no grupo exposto ao tabagismo, resistência à ação da insulina e maiores picos glicêmicos quando comparados aos controles, sugerindo que os fumantes têm maior dificuldade na captação de glicose, devido à menor disponibilidade de insulina, ao menor número e/ou funcionamento dos receptores de insulina ou à menor capacidade de indução da cascata de sinalização intracelular, que facilita a captação de glicose.

Como resultado desses fatores, os pesos e as dimensões renais no presente trabalho foram influenciados, culminando em volumes renais menores, fato também observado no estudo experimental de Dündar et al.^(19)^ No entanto, embora certas alterações morfológicas possam não significar comprometimento das funções renais, essa associação parece seguir de maneira diretamente proporcional no tabagismo, pois, ao se avaliar a espessura cortical do GT, foi observada atrofia significativa. De fato, a relação entre espessura cortical e função renal tem sido sugerida, especialmente quando há redução proporcional dos glomérulos,^(20)^ também observados no presente trabalho, com significativa redução das dimensões do diâmetro do corpúsculo renal, mas o espaço capsular aumentou no GT, com maior tempo de exposição, provavelmente devido às reduções do diâmetro glomerular, possibilitando a consequente diminuição na taxa de filtração glomerular (TFG).^(19)^

Na avaliação da densidade do volume mesangial, houve aumento significativo nos animais do GT. As células mesangiais que auxiliam e modulam a TFG e a área de superfície do glomérulo, graças à sua capacidade de contrair e relaxar em resposta a drogas vasoativas, podem sofrer significativa proliferação, com esclerose glomerular e fibrose túbulo-intersticial nos animais em resposta às lesões endoteliais glomerulares causadas pelo tabagismo.^(21,22)^

Devido às alterações morfológicas observadas, bem como um menor consumo de água pelos animais do GT, observado também nos estudos de Gonçalves-Silva et al.,^(23)^ os parâmetros bioquímicos das análises sanguínea e urinária também alteraram no GT, fornecendo subsídios para compreender os possíveis danos na função renal, já que os níveis de creatinina, creatinúria e volume urinário, utilizados para determinar a taxa de depuração de creatinina para se estimar a TFG, foi significativamente menor. O tabagismo promove aumento significativo na relação albumina-creatinina, com alterações na pressão capilar glomerular e na função renal,^(24)^ e são poucos os estudos que correlacionam a nicotina presente na corrente sanguínea dos tabagistas passivos com as doenças renais, principalmente quanto à exposição aguda.^(25)^

Com isso, a ação aguda da nicotina circulante pode estar relacionada à disfunção endotelial glomerular, já que os níveis persistentes de microalbuminúria, significativamente elevados no presente trabalho, determinam e classificam a instalação e a progressão da nefropatia e expressam fator de risco de disfunção e lesão endotelial na macro e na microcirculação.^(26)^ Cooper^(27)^ demonstrou que a nicotina acelera as nefropatias, as quais são detectadas por meio do aumento da microalbuminúria com progressão para proteinúria, concluindo que o tabagismo ativo e o passivo são tóxicos para a função renal.

## CONCLUSÃO

A exposição e a inalação passiva da fumaça do cigarro pelos animais ocasionaram alterações morfológicas e estruturais em seus rins. Somadas às alterações dos parâmetros bioquímicos por conta da exposição, o resultado foi uma diminuição da taxa de filtração glomerular, demonstrando que a exposição ao tabagismo passivo pode comprometer a função renal, com efeitos semelhantes aos do tabagismo ativo.
